# Fluorodeoxyglucose positron emission tomography/computed tomography findings after percutaneous cryoablation of early breast cancer

**DOI:** 10.1186/s40644-020-00325-y

**Published:** 2020-07-16

**Authors:** Takuya Adachi, Youichi Machida, Eisuke Fukuma, Ukihide Tateishi

**Affiliations:** 1 Department of Radiology, Tokyo Medical and Dental University, 1-5-45 Yushima, Bunkyo-ku, Tokyo, 113-8519 Japan; 2 Department of Radiology, Kameda Medical Center, Chiba, Japan; 3 Department of Breast Center, Kameda Medical Center, Chiba, Japan

**Keywords:** Cryoablation, Cryotherapy, Breast cancer, PET/CT

## Abstract

**Background:**

To document ^18^F-Fluorodeoxyglucose positron emission tomography/computed tomography (PET/CT) findings after percutaneous cryoablation for early breast cancer.

**Methods:**

Data of 193 consecutive patients who had undergone cryoablation for invasive ductal carcinoma or ductal carcinoma in situ ≤ 15 mm without a history of ipsilateral breast cancer, synchronous ipsilateral lesion, and with estrogen receptor positive/human epidermal growth factor 2 negative were enrolled. The imaging characteristics of the treated areas were evaluated and classified on CT images as one of two types: fatty mass or non-fatty mass type. The maximum standardized uptake value (SUVmax) of the initial post-cryoablation PET/CT, the CT type of the treated area and selected clinical factors (age, menopausal status, lesion area, breast density, timing of PET/CT) were retrospectively evaluated.

**Results:**

The median interval between cryoablation and the initial post-cryoablation PET/CT was 12 months. The median SUVmax of the treated area was 1.36. The CT findings of the treated area were classified as fatty mass type (*n* = 137, 71.0%) or non-fatty mass type (*n* = 56, 29.0%). The treated areas of patients with lower breast density, of older age, post-menopausal status, and lower radiation dose were significantly more likely to be of fatty mass type (*P* < 0.001). Non-fatty mass type averaged a significantly higher SUVmax than did fatty mass type.

**Conclusions:**

Post-cryoablation PET/CT findings are of fatty or non-fatty mass type. A non-fatty appearance, which can show higher SUVmax, does not necessarily denote recurrence.

## Background

Cryoablation, a minimally invasive treatment option for breast cancer, has been proposed in recent years because it causes minimal pain and has good esthetic outcomes [1]. Cryoablation is widely accepted as the treatment for tumors in several organs (kidney, prostate, liver) [2–4]. Increasing numbers of studies have reported the feasibility and short-time outcomes of cryoablation of early breast cancer [5–9]. Additionally, two clinical phase-2 trials (NCT01992250, NCT02200705) to investigate complete response and local recurrence rates after cryoablation of early breast cancer are ongoing.

^18^F-Fluorodeoxyglucose (FDG) positron emission tomography/computed tomography (PET/CT) has shown usefulness in detecting extraaxillary nodal involvement and distant metastases and may be the first whole-body imaging modality for restaging patients who are suspected of, or known to have, recurrence [10]. Although detection of residual tumor and recurrence after breast cryoablation is crucial, there are few published studies of imaging findings post-breast cryoablation [11].

The purpose of the present study was to document PET/CT findings after cryoablation for early breast cancer.

## Methods

The Institutional Review Board of our institution approved this retrospective study and waived the need to written informed consent for inclusion because this was a retrospective study using imaging data that had already been acquired. We declare that all human studies were performed in accordance with the ethical standards laid down in the 1964 Helsinki declaration and its later amendments.

### Patients

From July 2006 to December 2017, 273 consecutive patients underwent cryoablation for primary breast cancer in our institution. The indications for cryoablation were invasive carcinoma (IDC) or ductal carcinoma in situ (DCIS), no history of ipsilateral breast cancer, largest tumor diameter no more than 15 mm on preoperative imaging (mammography, ultrasound and MRI), no synchronous ipsilateral lesions, and estrogen receptor positive/human epidermal growth factor 2 (HER2) negative by immunohistochemical analysis of biopsy specimens in cases of IDC. Patients who had subsequently undergone resection, had not received adjuvant radiation therapy, had not undergone ^18^F-FDG PET/CT after cryoablation, whose initial postcryoablation PET/CT was performed more than 3 years after cryoablation, or who had undergone postcryoablation PET/CT in other institutions were excluded. The resultant study cohort comprised 193 patients.

### Cryoablation

Cryoablations were performed with one of the following two treatment systems: Visica I (Sanarus Medical, Pleasanton, CA, USA from October 2006 to April 2012) or IceSense 3 (IceCure Medical, Collierville, TE, USA from May 2012 to October 2014). The Visica I system uses argon gas as the cryogen whereas IceSense 3 uses liquid nitrogen.

All ablation procedures were performed using one cryoprobe with ultrasound guidance. After local anesthesia with 1% lidocaine containing epinephrine, the center of the target lesion was punctured with the cryoprobe and its tip advanced to the midpoint of the lesion. The cryoablation protocol was composed of an initial freezing, thawing, a second freezing, and warming. The durations of first freezing, thawing, and second freezing were each 10 min with Visica I, whereas they were 9, 8, and 9 min, respectively, with IceSense 3. Before and during cryoablation, normal saline was injected into the gap between the skin and subcutaneous fascia to prevent frostbite of the skin. At the end of the treatment, active warming was performed to enable safe removal of the cryoprobe.

### Radiation therapy

All study patients received irradiation after breast cryoablation according to the protocol for radiation therapy after lumpectomy. Whole breast was irradiated with two or three tangential fields. Some patients received a boost to the tumor bed with an orthovoltage unit of electrons.

### Pet/CT

All PET/CT studies were performed with one of the following two combined PET/CT systems: Discovery ST Elite Performance (GE Medical Systems, Milwaukee, WI, USA; *n* = 125; 64.8%) or Discovery IQ ODYSSEY 5R (GE Medical Systems, Milwaukee, WI, USA; *n* = 68; 35.2%). The patients fasted for at least 4 h before the PET/CT study, which started approximately 1 h after intravenous injection of 4.3 megabecquerels (MBq)/kilograms (kg) body weight of FDG. Patients rested during the time between injection and the start of PET/CT. The technical parameters for computed tomography (CT) included 50–150 mA with smart MA setting, 120 kVp, section thickness 5 mm, a gantry rotation time 0.5 s per tube rotation, and 512 × 512 matrix. After CT, whole-body PET emission scan was obtained with an acquisition time of 150 s per bed position and the matrix of 128 × 128 in Discovery ST and 192 × 192 in Discovery IQ.

### Imaging analysis

**Fig. 1:**
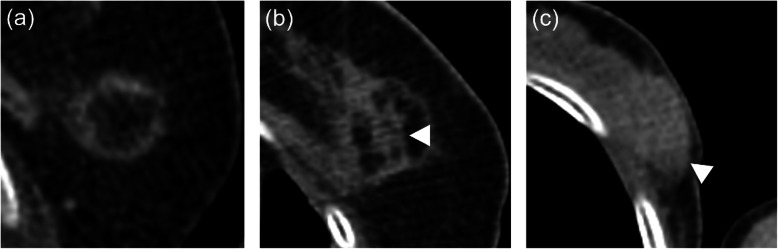
Classification of CT findings on ^18^F-FDG PET/CT after breast-cryoablation. CT findings were classified as fatty mass type (**a**, **b**) or non-fatty mass type (**c**). Fatty mass type was defined as the treated area including fat density, including a fatty mass with a soft tissue density septum (**b**, arrowhead). Non-fatty mass type was defined as the treated area consisting only of soft tissue density (**c**, arrowhead)

The initial post-cryoablation PET/CTs of all patients were retrospectively examined by two radiologists (T.A. with 3 years of experience in PET/CT imaging, U.T. with 20 years of experience in PET/CT imaging) for this study. A spherical volume of interest was manually located in the post-treatment area, and the maximum standardized uptake value (SUVmax) obtained at a workstation (Xeleris Functional Imaging Workstation, GE Medical Systems). The SUV was calculated according to the following formula: SUV = C/(ID/W), where C represents the decay-corrected tissue activity concentration in MBq/kg measured with PET, ID the injected dose in MBq, and W patient body weight in kg. The imaging characteristics of the treated areas were evaluated in each CT image and classified as one of two types: fatty mass or non-fatty mass. Fatty mass type was defined as the treated area having a fat density component and non-fatty mass type as the treated area consisting only of soft tissue. Treated areas that contained both fat density and soft density areas were classified as of fatty mass type in this study (Fig. 1, Image b). To identify correlations between PET/CT findings and patients’ clinical characteristics, age, menopausal status, lesion area, background breast density, number of days between cryoablation and PET/CT scan, radiation dose, and end date of radiation therapy were collected from clinical records. Breast density information was obtained from each patient’s preoperative breast MRI report.

### Statistical analysis

EZR software version 1.31 (Saitama Medical Center, Jichi Medical University, Saitama, Japan), a graphical user interface for the R software package, was used for all statistical analyses in this study [12]. The relationships between PET/CT findings, lesion area, and breast density were assessed by using Fisher’s exact test. The Mann–Whitney *U* test was used for continuous variables such as SUV, age, and number of days between cryoablation and PET/CT scan. *p* < 0.05 was defined as indicating a statistically significant difference.

## Results

**Fig. 2:**
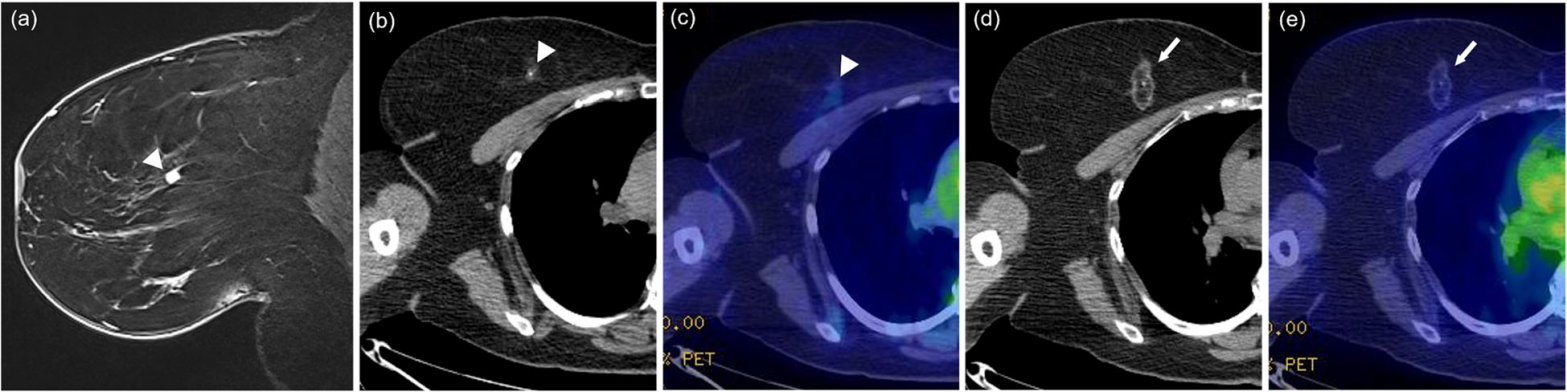
Fatty mass type. A 55-year-old woman with invasive ductal carcinoma. Breast magnetic resonance imaging (MRI) before cryoablation (**a**, arrowhead) showing a solid nodule of diameter 8 mm in the inner-upper quadrant of the right breast. The pre-cryoablation CT part of PET/CT (**b**, arrowhead) does not clearly show the breast cancer lesion identified by MRI. The pre-cryoablation fusion image of PET/CT (**c**, arrowhead) shows subtle FDG uptake (SUVmax, 0.89). On the CT portion (**d**, arrow) and (**e**, arrow) of the fusion image of PET/CT that was obtained 36 months after cryoablation, the treated area shows a focal fatty area surrounded by a rim like soft tissue density with subtle FDG uptake (SUVmax, 0.82). The calcification at the center of the treated area was present prior to cryoablation

**Fig. 3:**
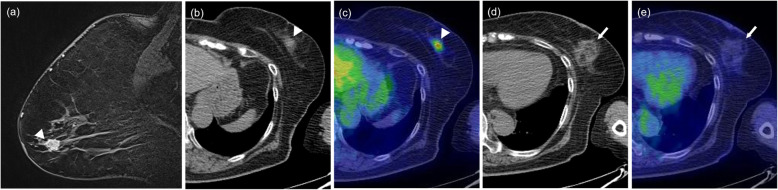
Fatty mass type. A 74-year-old woman with invasive ductal carcinoma. Breast MRI before cryoablation (**a**, arrowhead) showing a solid nodule of diameter 11 mm in the outer-lower quadrant of the left breast. The pre-cryoablation CT portion (**b**, arrowhead) and fusion image (**c**, arrowhead) of PET/CT show a solid nodule with abnormal FDG uptake (SUVmax, 3.53). On the CT portion (**d**, arrow) and (**e**, arrow) fusion image of PET/CT that was obtained 11 months after cryoablation, the treated area shows a focal fatty area and septa of soft tissue density with subtle FDG uptake (SUVmax, 1.34)

**Fig. 4:**
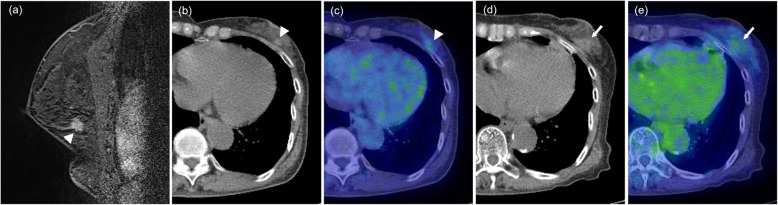
Non-fatty mass. A 73-year-old woman with invasive ductal carcinoma. Breast MRI before cryoablation (**a**, arrowhead) showing an irregularly shaped nodule of diameter 11 mm in the outer-lower quadrant of the left breast. The pre-cryoablation CT portion (**b**, arrowhead) and fusion image (**c**, arrowhead) of PET/CT show a solid nodule with abnormal FDG uptake (SUVmax, 1.78). The CT portion (**d**, arrow) and (**e**, arrow) fusion image of PET/CT that was obtained 11 months after cryoablation shows a non-fatty mass and subtle FDG uptake in the treated area (SUVmax, 1.86)

The patients’ basic characteristics are summarized in Table 1. The initial post-cryoablation PET/CTs were performed a median of 12 months (range, 2–36 months) after breast cryoablation. Cryoablation was performed with DCIS in 29 patients (15.0%) and with IDC in 164 patients (85.0%). The median SUVmax on PET/CT was 1.36 (range, 0.56–2.32). Typical PET/CT findings after breast cryoablation are shown in Figs. 2, 3 (fatty mass type) and Fig. 4 (non-fatty mass type).

**Table 1:** Clinical data of all patients in this study

Clinical data	(*n* = 193)
Age (years), median (range)	57 (33–82)
Size (mm), median (range)	9 (2.5–15)
Pathology (IDC), n (%)	164 (85.0)
Lesion in right breast, n (%)	105 (54.4)
Duration between cryoablation and initial post-cryoablation PET/CT (months), median (range)	12 (2–36)
Radiation dose (Gy), median (range)	47.7 (39.6–60)
Boost to tumor bed, n (%)	42 (21.8)
Boost to tumor bed (Gy), median (range)	9 (2.5–12)
Duration between radiation and initial post-cryoablation PET/CT (months), median (range)	10 (3–33)

*IDC* Invasive ductal carcinoma, *PET/CT* Positron emission tomography/computed tomography

The relationships between CT findings after cryoablation and patients’ clinical factors, details of radiation therapy, and SUVmax on initial post-cryoablation PET/CT are shown in Table 2. Additionally, the relationship between section of the breast affected by the lesion and PET/CT findings was assessed by classifying locations (upper vs. lower, inner vs. outer). Patients with lower breast density, older age, and post-menopausal status were significantly more likely to show fatty mass type on the initial post-cryoablation PET/CT (*p* < 0.001). The non-fatty mass type, which was identified in 56 patients (29.0%), showed significantly higher SUVmax than the fatty mass type (*p* < 0.001). Section of lesion, radiation dose including any boost, interval between cryoablation and initial post-cryoablation PET/CT, and interval between radiation therapy and initial post-cryoablation PET/CT did not differ significantly between the two types of CT findings. Calcification was seen in the treated area on CT in 7 of 193 patients (3.62%). Patients with calcification in the treated area had undergone PET/CT 12–36 months (median, 21 months) after breast cryoablation. PET/CTs with calcification in the treated area were performed significantly later than those without calcification (*p* = 0.003).

**Table 2:** Relationship between PET/CT findings and clinical characteristics

		CT findings		
		Fatty mass type (*n* = 137)	Non-fatty mass type (*n* = 56)	*p* value
Age (years), median (range)		62 (38–82)	49 (33–77)	< 0.001
Postmenopausal, n (%)		103 (75.7)	20 (37.7)	< 0.001
	NA	1	3	
Breast density, n (%)	BIRADs A	19 (13.9)	1 (1.8)	< 0.001
	B	55 (40.1)	5 (8.9)	
	C	49 (35.8)	17 (30.4)	
	D	14 (10.2)	33 (58.9)	
Lesion size (mm), median (range)		9 (2.5–14.0)	9 (4.0–15.0)	0.368
Location of lesion in breast, n (%)	Upper	94 (78.0)	37 (71.2)	0.439
	Lower	27 (22.0)	15 (28.8)	
	Inner	34 (31.2)	14 (27.5)	0.713
	Outer	75 (68.8)	37 (72.5)	
Duration between radiation therapy and initial post-cryoablation PET/CT (months), median (range)		10 (4–33)	10 (3–26)	0.744
Radiation dose (Gy), median (range)		44.0 (39.6–50.0)	47.0 (40.0–50.25)	0.697
Boost to tumor bed, n (%)		24	18	0.032
Boost to tumor bed (Gy), median (range)		9 (8.1–12.0)	9 (2.5–10)	0.719
Duration between cryoablation and initial post-cryoablation PET/CT (months), median (range)		12.0 (1–36)	12.5 (7–29)	0.743
SUVmax, median (range)		1.30 (0.56–2.32)	1.49 (0.89–2.24)	< 0.001
Size of the post-cyroablation change on PET/CT (mm), median (range)		28 (12–44)	25 (11–43)	0.002

*PET/CT* Positron emission tomography/computed tomography, *NA* Not available; Breast density BIRADs A, fatty; BIRADs B, scattered fibroglandular density; BIRADs C, heterogeneously dense; BIRADs D, extremely dense

Lesions located in a boundary zone, such as lesions level with the nipple in cephalocaudal height, were excluded from analysis of locations of lesions (Upper or Lower, Inner or Outer)

During this study period, there was one (1/193, 0.5%) patient who developed a recurrence after the treatment of invasive carcinoma in the lower-inner quardrant of the left breast. She had showed 10 mm in the largest diameter before treatment at the age of 55. Four months after cryoablation, an irregular-shaped mass in the left breast was observed on breast MRI and the recurrence was confirmed by open biopsy; the recurrent lesion was not visible on the initial post-cryoablation PET/CT.

## Discussion

This study investigated ^18^F-FDG PET/CT findings after breast cryoablation and the relationships to SUVmax and patients’ characteristics. We found that non-fatty mass type was related to higher SUVmax.

The ratio of fatty mass type in patients with higher breast density was relatively low because fat necrosis can result from fatty breast tissue. A previous study reported that breast density decreases with age [13]. This tendency is consistent with our finding that aged or postmenopausal patients had a significantly higher ratio of fatty mass type. Additionally, a significantly higher proportion of patients who received boost irradiation had the non-fatty mass type. We attributed this finding to the fact that boost irradiation is mainly administered to younger patients in accordance with the strategy for radiation therapy after lumpectomy.

Some patients showed calcification on their initial post-cryoablation PET/CTs. They tended to have undergone PET/CT later than those without calcification and the calcification seemed to be dystrophic.

During this research period, only one patient developed local recurrence which could not be detected on PET/CT. As a matter of fact, our population showed good course as natural outcome, because we analyzed PET/CT of early-stage breast cancers.

Only one study has reported imaging findings after breast cryoablation [11]. In that study, suspicious enhancement on breast MRI after cryoablation was observed in 7 of 54 patients (13.0%); however, the shape of the treated area was not reported. To the best of our knowledge, our study is the first to describe the metabolic and morphologic findings of treated areas of breast cryoablation.

PET/CT findings after cryoablation for early breast cancer reveal two components: fat density and soft tissue density components. A previous opinion article reported that cryoablation causes both direct and indirect cell injury that results in coagulative necrosis or apoptosis [14]. Mieczyslaw et al. reported the pathological findings after cryoablation for primary breast cancers that were resected between Days 1 and 35 after cryotherapy [15]. They reported that histopathological examination of the treated area showed fibrosis, granulation, inflammatory infiltrate, and focal scarring 5 weeks after cryoablation and fat necrosis 4 to 7 days after cryoablation. Therefore, the fat density and soft tissue density components on post-cryoablation PET/CTs correspond to fat necrosis and post-necrotizing changes (fibrosis, granulation, scarring, inflammatory infiltrate), respectively.

It is known that CT values affect SUVmax in CT-based attenuation correction on PET/CT. CT-based attenuation correction could increase SUVmax of non-fatty mass more than that of fatty mass. In addition, the details of radiation therapy can affect PET/CT findings after breast-cryoablation because soft tissue injury caused by radiation therapy is associated with inflammation in the treated area [16].

Although non-fatty mass type, which shows higher SUVmax, can mimic recurrence on ^18^F-FDG PET/CT this finding does not indicate relapse.

Possible limitations of this study are as follows. First, because of its retrospective nature, the interval between cryoablation and PET/CT was not uniform. PET/CT findings can differ between follow up phases. Our findings should be investigated in prospective trials with strict timing of follow up. Second, the two PET/CT machines used in this study had different specifications, which may have affected SUVmax. Third, we did not assess the usefulness of PET/CT in detecting recurrence because only one study patient relapsed during follow up. Although several studies have shown that postoperative PET/CT in breast cancer performs better than conventional imaging (whole body CT and bone scanning, US, mammography) when recurrence is suspected on the basis of clinical findings or biological markers [17–25], additional large studies with enough recurrences are necessary for investigating the optimal modality for restaging after breast cryoablation.

## Conclusions

We found that there are two types of CT findings on ^18^F-FDG PET/CT after cryoablation for early breast cancer and that breast density affects post-cryoablation CT findings. The non-fatty mass type, which shows higher SUVmax, does not necessarily indicate relapse.

## Data Availability

The datasets during and/or analysed during the current study available from the corresponding author on reasonable request.

## Abbreviations

^18^F-FDGPET/CT: ^18^F-Fluorodeoxyglucose positron emission tomography/computed tomography
SUVmax: The maximum standardized uptake value
IDC: Invasive carcinoma
DCIS: Ductal carcinoma in situ
HER2: Estrogen receptor positive/human epidermal growth factor 2
MBq: Megabecquerels
